# The Treatment of Long Standing Pleural Effusion—Caries of the Vertebræ with Phthisis—An Example of Extreme Emaciation

**Published:** 1883-12

**Authors:** William Pepper

**Affiliations:** Professor of Clinical Medicine in the University of Pennsylvania


### BUFFALO

### Medical and Surgical Journal.

Vol. XXIII. DECEMBER, 1883. No. 5.

### Original Communications.

### The Treatment of Long Standing Pleural Effusion—Caries of the Vertebræ with Phthisis—An Example of Extreme Emaciation.

A CLINICAL LECTURE DELIVERED AT THE HOSPITAL OF THE UNIVERSITY OF PENNSYLVANIA, MAY 19TH, 1883.

By William Pepper, M. D., LL. D.
Professor of Clinical Medicine in the University of Pennsylvania.
Reported by William H. Morrison, M. D.

Gentlemen—I again bring before you the colored man whom you saw two weeks ago. You will remember that he had a large effusion in the right pleural cavity which had lasted four months and was the result of an attack of acute pleurisy. The left chest was almost entirely filled by the liquid, the diaphragm was pushed downwards and the heart was pushed to the right so that its apex beat was felt under the sternum. I told you that we should try to remove this effusion by the process of absorption. Absorption of this liquid, of course, had to take place from the surface and through the vessels of the pleura, and upon the extent to which that surface and those vessels were changed would depend the ease or difficulty with which absorption would occur. In a mild attack of acute pleurisy, the inflammation subsides, the congestion is relieved by the effusion and the fever passes away. If the patient is kept in bed, absorption takes place spontaneously and in a few weeks he is well. If the inflammation be more severe and attended by the formation of a large amount of plastic lymph, covering the vessels and the absorbent surface, the effuse serum will no more be absorbed than if it were in a leather bottle. It may remain serous, or from a renewal of the inflammation in the feebly organized membrane, become mixed with pus, which may remain for months or years. Between these two extremes there is every degree of capacity on the part of nature for the removal of pleural effusion. Hence, in making our prognosis in these cases, there is always an element of uncertainty, viz., the state of the pleura. If the liquid is purulent we cannot hope to have it absorbed; but so long as it remains serous we have a right to hope for its removal in this way. The question whether or not it will be absorbed will depend upon the state of the pleura, which cannot be determined by external examinations. The longer a case has lasted, the less is the chance of having it removed by nature, and the more reason is there to think that the state of the pleura will not admit of absorption. Too much time should not be wasted in endeavoring to promote absorption, but before resorting to aspiration the patient should be given a reasonable chance.

The best plans of treatment for chronic inflammatory hydrothorax seems to me to be one of the two following: In the first place, the use of free blistering to the surface of the chest by means of blisters six inches square, repeated every couple of weeks, and internally the administration of iodide of potassium and digitalis, to which may be added acetate of potassium in such doses as are readily tolerated by the system. If there is no fever, the patient may take moderate exercise and should have a light dry diet which will promote the absorption of the liquid.

The second plan of treatment is by the free sweating with jaborandi conjoined with rest and restricted diet.

I told you that in this case we should try the latter of these two plans. On entering the hospital two weeks ago he weighed 184 pounds; to-day he weighs 168 pounds; he has, therefore, lost 16 pounds in fourteen days. During one sweat, which lasted three hours, he lost two pounds in weight, which would indicate a loss of one quart of liquid. He was at first put on the use of small doses of jaborandi—sixteen drops thrice daily—his diet at the same time being restricted to four ounces of milk every two hours. This cut his weight down in one week ten pounds, but did not produce much effect upon the pleural effusion. This made me fear that the pleura was so thickened that the liquid could not be absorbed. Last week I determined to try the effect of a more vigorous impression of jaborandi. The milk was increased to six ounces every two hours and he was allowed a small piece of bread three times a day. During the past week he has received one drachm of the fluid extract of jaborandi every other day. As a result he has lost six pounds in weight. Between the jaborandi sweats he has gained in weight. I should state that the urine has been examined but contains no albumen nor tube-casts. Its specific gravity is 1018 and the quantity is from four to six pints per day.

It remains now to determine what effect this has had upon the pleural effusion. His breathing is much easier than it was last week, and he feels very comfortable. The heart's action is still rather rapid and weak. The apex beat has returned towards the left and is now at the left edge of the sternum. It is still an inch and a half too far to the right. There has been a considerable decrease in the height of the liquid. Dullness is now reached at the fifth rib. The stomach yields its tympany some distance above the border of the ribs, showing that the diaphragm has risen. At the last examination the diaphragm was pushed down, and the resonance of the stomach was below the border of the ribs. The height of the diaphragm is as important as the upper line of dullness in determining the amount of effusion. The upper line of dullness may not be very high, and yet if the diaphragm be pushed, a large amount of liquid may be present.

There is good respiratory murmur to the level of the liquid. Below that it is feeble, distant and lost. There has been, then, during the past week, a marked reduction in the amount of the effusion. I think that at least a quart of fluid has been removed. This would equal two pounds, and the remaining of four pounds must have been due to loss of solid material.

It remains to determine what course of treatment shall be pursued to remove the rest of the effusion. I am disposed to suspend operations for a week and allow nature to do what she can. Having started the process of absorption, and having the vessels well drained, I shall put him on the use of iodide of potassium, giving five grains three times a day. I shall also allow him a more liberal diet, an egg at breakfast, meat at dinner, and bread and butter three times a day. If this does not remove the liquid, I shall return to the use of jaborandi, giving him one or two more free sweats, and if this is not successful, I shall aspirate. If at the end of the week I find that the effusion has diminished, I shall continue the treatment which I have suggested, but if the quantity of liquid has increased, I shall immediately aspirate.

In cases of this kind we have to consider whether we are justified in adopting the severe treatment which was pursued in this case instead of tapping. Even if tapping is adopted there is danger that the effusion will immediately re-accumulate; while by following the plan of treatment which was employed in this case, not only is the effusion removed, but the pleura is brought into a healthier condition and the liability to return is lessened. I think that in this case the treatment was entirely justifiable, but I do not think that we should carry it further at the present time.

### CARIES OF THE VERTEBRÆ WITH PHTHISIS.

This man has been working in the gas house and exposed to great changes of temperature and to a malarial atmosphere. After working in the gas house and becoming very hot, he would go out into the night air, with nothing on but his drawers and shirt, and sleep on the ground. He began to suffer with severe pain over the right short ribs. This pain was made worse by exercise and exertion and by walking. He also had a cough and muco-purulent expectoration, but the pain was so bad that he did not refer to the cough when he first applied for relief.

At that time it was thought that the pain was due to intercostal neuralgia, the result of malaria, but it did not yield to quinia and arsenic. We then thought that it might result from a strain, but there was no history of any injury of that kind. The pain was paroxysmal in character and very much influenced by certain positions. It was much worse when he laid on his back or the left side, and the only way that he could sleep with comfort was on the right side. This mechanical feature of the pain was very well marked. When at last he spoke of the cough, we examined the lungs and found phthisis of the left upper lobe. There was solidification with some softening in this region. On stripping him, I found that the curvature of the spine was excessive. Although I cannot say that this curvature has taken place recently, yet it is so marked as to suggest, when taken in connection with the disease of the lung, and the persistent pain, that there may be some morbid condition of the anterior faces of the bodies of the vertebræ in the lower dorsal region. The diagnosis at which I arrived was that this man was suffering from incipient caries or subacute inflammation of the cartilages and periosteum of the bodies of the vertebræ, in the lower dorsal region, associated with phthisis of the left upper lobe of the lung. Ten days ago I applied the actual cautery at the level of the pain and had a plaster of Paris jacket applied. He states that the pain has been a great deal better, that he sleeps better and is able to walk better since it has been applied. Having obtained so much benefit from the plaster of Paris jacket, I shall put him to the expense of having a rawhide jacket made, so that the treatment of the dorsal region by cauterization may be continued. The lesion in the lung is not advanced enough, nor advancing rapidly enough to make it otherwise than proper that we should try to give this man as much relief as possible; and I think it will pay him to have a jacket that he can wear all the time without renewing it every few months.

### AN EXAMPLE OF EXTREME EMACIATION, THE RESULT OF SUCCESSIVE ATTACKS OF ACUTE DISEASE.

The next patient is a young girl who is brought from the country with the history of a series of severe illnesses which have caused great weakness and emaciation, which is now complicated by contraction of the lower limbs. The following is her history: L. L., age 14 years; a school girl; has never had any hard work to do, and has never been especially exposed. She was never very strong, and was troubled with a weak stomach. She would suffer from dyspepsia after the least indiscretion in diet, and was liable to attacks of sick headache. Two years ago she was in bed for six weeks with "a bad head and stomach." She has had the various specific diseases of childhood. Five months ago she had a severe attack of diphtheria, and to give you an idea of the severity of this form of diphtheria, I may state that she had a brother and sister die in the same house in which she was sick. While convalescing from diphtheria she was attacked with pneumonia of the lower lobe of the left lung. This made her very ill, and again reduced her extremely low. When she began to improve from the pneumonia, she got typhoid fever, and has gone through a long and tedious spell of this disease. She is now convalescing from this last disease.

Her present weight is forty-nine pounds; as she is of the average height for her age, I judge that she must have lost more than fifty per cent. of her whole weight. She has bed sores over the sacrum and over the left hip. The tendons of the legs and of the fingers of the right hand are contracted, but there is no anchylosis. Her appetite is fair, the digestion good and the bowels regular. If she makes any exertion, she suffers from shortness of breath. She also has spells of dyspnœa. The lungs seem to be healthy. The pulse is rapid, but there is no cardiac murmur.

Dr. Wm. H. Hughes has examined the blood and reports that there are 3,500,000 red corpuscles in the cubic millimetre, instead of 5,000,000, as there should be. There is one white corpuscle to every 850 red. There is, therefore, decided anæmia. There are also a large number of imperfectly developed red globules.

The emaciation is extreme. Such a degree of emaciation is, fortunately, very rare. The skin is harsh and dry from neglect, as well as from the removal of the adipose tissue by absorption. The skin over the bony processes is drawn so tight and thin that it seems to be almost transparent. In places, there are bands in the derm, giving a cicatricial appearance to the skin. A little more and we should have had ulceration over the convexity of the knees. This is a result of bad nursing. The limbs have been allowed to remain in a flexed condition, the skin has become congested and inflamed, and the derm has been torn, giving rise to these bands of cicatricial tissue.

As a consequence of long disuse and atrophy of the muscles, contraction of the limbs has occurred, so that any attempt to extend the legs causes severe pain. All this might have been prevented, by a proper exercise of the parts during the patient's illness. This is a most valuable lesson to us of the importance of attending to these points during prolonged and exhausting sickness. We have now to deal with a condition which is almost as serious as any that she has passed through, and which will be longer, more tedious and more painful in its management.

Otherwise the child is very well. The tongue is clean; the appetite is good and the bowels regular. I show you the bed sore on the left hip. There is here a fungous ulcer, two inches in diameter, almost laying bare the trochanter.

The treatment of this case will require great patience and gentleness. In the first place, she must be carefully nourished. She will be given milk, eggs, meat, porridge, bread and butter, beer if she will drink it, if not, milk punch; oil will be rubbed into the skin twice a day; as the inunctions are made, there will be manipulation of the muscles and passive movements of the joint. If the contractions are not overcome by systematic massage and passive movements; we shall have a Stromeyer splint applied, and by this means gradually and gently overcome the contraction. Nothing like violence or haste is allowable in a case of this kind. Such a course would immediately excite inflammation in these feeble tissues and ulceration would follow.

This treatment shall be carried out, and I hope, in a few weeks, to be able to show her to you greatly improved.

